# Supplementary material for: Exploring the workload of informal caregiving in the context of HIV/NCD multimorbidity in South Africa
Source: PLOS Glob Public Health. 2024 Oct 8;4(10):e0003782. doi: 10.1371/journal.pgph.0003782 (PMC11460685; doi:10.1371/journal.pgph.0003782)
Supplement: S1 Text — (DOCX) [file pgph.0003782.s002.docx]

1. Affective state

| ***Main Question:*** *How has having more than one health condition or illness affected you emotionally?* |  |
| --- | --- |

Extra prompts for patients

| - When you got the diagnosis of your first & second (or third) condition, how did this make you feel? And can tell you me a bit more about when you were diagnosed with your conditions (medical history) |  |
| --- | --- |
| - What condition do you find harder to live with, emotionally? |  |
| - How has having multiple chronic conditions changed your life? (physically, emotionally, economically) Has it changed you as a person or your relationships? Has it changed the way you live your life, limited you in any way? If yes, please explain how…. |  |
| - Has having multiple conditions changed the way you see yourself or think about yourself? |  |
| - Have you ever experienced negative attitudes (stigma) from others? And how did you react to this? |  |
| - How do you feel about having these conditions (positive/negative emotions)? Do you ever feel depressed when you are thinking about your conditions? |  |

1. Adaptation to disruption

| ***Main question:*** *How has having more chronic conditions changed your life (physical & psychological effects; limitations on way of life, work, and routines; impact on relationships; family life)?* |  |
| --- | --- |

Extra prompts for the patient:

| - How is it to live with two or more chronic conditions physically and emotionally?? And how are you coping with the changes to your life? |  |
| --- | --- |
| - How easy or difficult has it been to accept your situation? How have you overcome some of the difficulties you have faced? |  |
| - What has helped you adapt to living with two or more chronic conditions? |  |
| - How has having more than one chronic condition changed your life (physical & psychological effects; limitations on way of life, work and routines) |  |
| - How do you manage work in your situation (time off for appointments and illness; attitude of co-workers and boss; limitations on ambitions?) |  |
| - What do you do to cope with your conditions? Are there particular strategies you use to stay healthy? (eg. a strict sleeping routine, having a pill box, counselling, joining a support club etc). |  |

Extra prompts for the carers

| - How has your caring role changed your life (eg: limited your freedom, changed your routine and relationships)? |  |
| --- | --- |
| - How have you adapted to these changes? |  |

1. Socio-economic status

| ***Main Question:*** *How do you think your age/gender/income affects your health (physical; mental) and your experience of health care?* |  |
| --- | --- |

Extra prompts for patients

| - How do you think your age or being male or female affects your health and the care you get from the clinic? (your health can be physical or psychological/emotional). |  |
| --- | --- |
| - And how do you think how much money you have affects your health and your (access to) care? For example, is it easy to pay for medication, special food, or aids that you need for your conditions? |  |
| - Are there things that you need to take care of your health that you cannot afford? If yes, can you tell me what these are? |  |

Prompts for carers

| - What are your thoughts about the money that the patient needs to spend to keep healthy? health needs? (consider time, diet, transport etc) - Do you think that age or gender influence the care the patient gets at the clinic/hospital? |  |
| --- | --- |

1. Spatial location of services

| ***Main Question:*** *What are some of difficulties you experience in getting to health services (clinic; hospital)?* |  |
| --- | --- |

Extra prompts for patients

| - How do you think time, work and family commitments and transport impacts your ability to get care (go to the clinic, get medication, etc)? |  |
| --- | --- |
| - How do you think the area in which you live influences your health and getting health and/or social cOare (crime; diet; physical activity; availability of community resources)? Can you also tell me how you are getting to the facilities? |  |
| - How do you deal with these problems (if present)? |  |

Extra prompts for the carers

| - What are the difficulties you, as the carer, experience in helping the patient get the health and/or social care they need from the clinic? |  |
| --- | --- |
| - What are your thoughts/feelings about these issues? |  |

1. System quality

| ***Main question****: What is your experience of the health services you use? What is good/not so good about them? What would you like to see improved/changed?* |  |
| --- | --- |

Extra prompts for patients

| - What do you think of the way the health services are organized?   - Opening times; waiting times; queue management; the frequency of visits; role of different health workers*;* location of pharmacy; referral to other doctors or the hospital; patient records   - The condition of facilities such as bathrooms, benches etc   - Do you feel safe/secure when visiting in the clinic?   - How do you think health services will change in the future (eg. Provision of health care, extra support, monetary support)? |  |
| --- | --- |
| - Once you are at the clinic, how easy is it to find out where you need to go, or which doctor or nurse you need to see? What difficulties do you experience when in the clinic? |  |

Extra prompts for carers

| - What has been your experience of the quality of health care when you go with the patient to the clinic?   - General services, admin, staff, staff attitude, waiting time etc.   - How do you think health services will change in the future (eg. Provision of health care, extra support, monetary support)? |  |
| --- | --- |

1. Interaction quality (Interaction with health workers)

| ***Main Question:*** *How would you describe your relationship with health workers?* |  |
| --- | --- |

Extra prompts for patients

| - Can you describe the relationship you have with your health worker(s)? What do you like about them, what can they do better or improve? |  |
| --- | --- |
| - How do you, as a patient, feel when you are interacting with them? |  |
| - How do your health workers help you manage your conditions? |  |
| - Has having more than one chronic condition changed anything in your relationship with health workers? |  |
| - How do you feel about decision making in relation to how to manage your health? |  |
| - What do you think would improve the quality of your interaction with your HCPs? |  |
| - Is there anything else you would like to share about your relations with your health workers? |  |

Extra prompts for the carers

| - Can you tell me more about your interaction with health worker(s) when you come with the patient to the clinic? |  |
| --- | --- |
| - Is there anything that they (the health workers) could improve to help you or the patient? |  |

1. Technological support

| ***Main Question:*** *How do you as a patient use technology (cell phones, aids, computers) to help you manage your conditions?* |  |
| --- | --- |

Extra prompts for patients

| - What do you know about technologies that can help you manage your conditions? Do you use any? Such as SMS reminders, apps, glucometer etc? |  |
| --- | --- |
| - Do you have any cellphone apps that remind you to take your medication? |  |
| - Do you have a glucose meter or blood pressure machine to help you? And yes, how often do you use it? |  |
| - Are you comfortable using the technologies available to you? Why do you find it easy or difficult? |  |
| - How does the technology you mentioned help manage your conditions? |  |
| - What other type or technology (such as app, SMSes etc) would you like to use? And how do you think this will help you? |  |

Extra prompts for the carer

| - Do you know of any technologies that could help you with your caring tasks? If yes, please describe. |  |
| --- | --- |
| - Do you have access to any of these technologies? And how would they help you? |  |

1. Cognitive advantage (health information)

| ***Main Question****: What do you understand about your conditions? How have you gained info/knowledge about your different conditions and how to take care of your health?* |  |
| --- | --- |

Extra prompts for patients

| - What kind of (health) information do you get about your conditions? For example through pamphlets, doctor’s advice, talks at health facilities, radio, TV shows, internet? |  |
| --- | --- |
| - Is this information relevant to you? Do you trust it? And is it useful? And is it easy to read? And is the information consistent or do you find it confusing? |  |
| - What would you say are your best sources of information? |  |
| - Do you have enough information? And what are you missing? |  |
| - What do you think you know enough about? And what would you like to know more about? |  |
| - If you have a particular symptom that is scary/concerning, where do you go to get extra information and support? |  |

Extra prompts for the carers

| - How did you learn more about the condition of the patient? Did you get pamphlets, speak to health worker, get information from radio/TV/internet? Was it useful? |  |
| --- | --- |
| - Did you receive any information specifically for your role as a carer? If yes, what kind of information is that? |  |
| - How well informed do you think you are about the condition? |  |

1. Caregiver support

| ***Main Question:*** *Who is helping/supporting you to manage your own healthcare?* |  |
| --- | --- |

Extra prompts for the patients

| - Can you please tell me more about the person who came with you to the interview. Is he/she your main caregiver? |  |
| --- | --- |
| - How do they help you in your daily routines? And what do you appreciate the most? Can you tell me more about your relationship? |  |
| - Do you do something to thank your carer for their service? And if yes, what do you do? |  |
| - How do you think that your carers combine supporting you with other tasks they need to do? For instance, do you think they have enough time to care for you, how do they cope emotionally and is the work hard? |  |
| - Is there anyone else in your network who assists you? |  |

Extra prompts for the carer

| - How do you cope with your care-giving role? How do you feel about it? |  |
| --- | --- |
| - Can you tell us more about your experience of care giving? Has it become harder/easier over time? And why? |  |

1. Help-seeking

| **Main Question:** *Have you ever had to get help for a health emergency? And how do you seek help when you have concerns about your health conditions? Can you tell me more?* |  |
| --- | --- |

Extra prompts for the patient

| - If you need help with managing one or more of your conditions, do you feel you can ask for help? Where do you get the help from? For example, do you speak to a counsellor, family member or neighbour? |  |
| --- | --- |
| - What do you do when you need urgent help with your condition? Who helps you make decisions in a health crisis (emergencies) (eg. Who speaks to the doctor/nurse, makes sure you get to the clinic) |  |

Extra prompts for the carer

| - Have you ever had to help the patient get help in a health crisis/emergency? If yes, what did you do? And how did it make you feel? |  |
| --- | --- |

1. Situated decision-making

| ***Main Question:*** *Do you think that you (as a patient) manage your conditions well? And can you explain why/why not?* |  |
| --- | --- |

Extra prompts for the patient

| - How easy or difficult do you find all the things you have to do to stay healthy (eg. Taking medications, diet, activities)? Is it stressful? And why? Which condition is the hardest to manage? |  |
| --- | --- |
| - How do you feel when things are out of control (eg. When you have symptoms and do not feel well)? |  |
| - How much control do you feel you have over your conditions? And can you explain why? |  |
| - And what helps to make you feel more in control of your health? Do you have particular strategies? |  |
| - What could make it easier to manage your conditions in future? |  |
| - Do you think the quality of health services will improve in the future? And what do you thinks need to be changed to make the services better? |  |

Extra prompts for the carer

| - How do you see your role as a carer in the future? |  |
| --- | --- |
| - How do you think health services will change in the future (eg. Provision of health care, extra support, monetary support)? |  |

1. Competence

| ***Main Question:*** *As a patient, how do you show the nurses/doctors or your carer that you are trying to stay healthy and do what you are supposed to do?* |  |
| --- | --- |

Extra prompts for the patient

| - What do you need to do to stay healthy? How often do you need to take medication, what foods do you need to eat, how often do you go for check-ups? What else? |  |
| --- | --- |
| - What tasks are you managing well/not so well, and why? What could help you manage certain tasks better? |  |
| - What tasks are easy for you to stay healthy? And what is difficult? And why are they hard/easy? What could help you manage certain tasks better? |  |

Extra prompts for the carer

| - What are your tasks as the main carer? |  |
| --- | --- |
| - What makes it hard to manage all these tasks? And what is easy? |  |
| - What could help you in your role? |  |

1. Covid-19 Questions

| ***Main Question:*** *How has COVID-19 impacted how you take care of yourself and your health?* |  |
| --- | --- |

Extra prompts for patient

| - Has COVID-19 made it easier or harder to stay healthy? And why? For example, has it been more difficult to get food, have you lost income, do you feel less safe, is it more difficult to get around? |  |
| --- | --- |
| - What precautions have you been taking to be safe when seeking health care? Have these been changes been easy or difficult? |  |

Extra prompts for the carer

| - How has COVID-19 changed your role as a carer? |  |
| --- | --- |
| What precautions have you been taking to ensure the safety of yourself and patient? |  |
| Has COVID-19 made it easier or harder for you to assist the patient? And why? |  |
