# Supplementary material for: Exploring the workload of informal caregiving in the context of HIV/NCD multimorbidity in South Africa
Source: PLOS Glob Public Health. 2024 Oct 8;4(10):e0003782. doi: 10.1371/journal.pgph.0003782 (PMC11460685; doi:10.1371/journal.pgph.0003782)
Supplement: Attachment — Submitted filename: Response to the reviewer 18032024 FINAL .docx [file pgph.0003782.s003.docx]

*Include when submitting revised manuscript:*

- *A rebuttal letter that responds to each point raised by the editor and reviewer(s). You should upload this letter as a separate file labeled 'Response to Reviewers'.*
- *A marked-up copy of your manuscript that highlights changes made to the original version. You should upload this as a separate file labeled 'Revised Manuscript with Track Changes'.*
- *An unmarked version of your revised paper without tracked changes. You should upload this as a separate file labeled 'Manuscript'.*

***Response to the reviewers:***

***Thank you so much for giving us the opportunity to review this manuscript and to give constructive feedback to the paper.***

***We have added the recommended changes to the text and responded in the table below. All small changes are done using the track changes, the rest is embedded in the text.***

***We hope that we have address all your concerns adequately.***

***Warm greetings,***

***Dr Myrna van Pinxteren & Ms Charlotte Slome, on behalf of the authorship team.***

| Reviewer 1 comments | | | | |
| --- | --- | --- | --- | --- |
| Comment | |  | Corrected/Revised | |
| 1. Spelling mistakes | 1a. ✅ | page 3, 3rd paragraph, line 4; ‘efnancial’ | Page 11, 457 | Corrected. |
|  | 1b. ✅ | page 12; omission--“….we need encourage participation…” last sentence of paragraph 6 under ‘discussion” | pg.15, 663 | Corrected. |
| 2. Study design, recruitment, and context | 2a. ✅ | While the study locations are well described and it is perfectly ok to have purposively select them; the authors need to explain what informed selecting those two study locations and even the study provinces out of 9 provinces and literally 1,001 communities that could have been selected. This is critically important to provide more scientificity to the study. | Pg. 4, Line 196 | We have provided more detail about the selection of the field-sites and the rationale to conduct comparative work.  *“Fieldwork for the EXTRA study was conducted in two locations in South Africa, the urban township of Gugulethu and rural Bulungula. We deliberately chose two comparative field-sites, as we wanted to explore the perceived treatment burden of PLWMM in both rural and urban settings, as access to care, support networks and caregiving experiences may differ. In Gugulethu, we selected participants who were enrolled in a linked cross-sectional study that measures the prevalence, patterns and factors of multimorbidity in adults with HIV (3). The field-site in Bulungula was chosen due to an ongoing relationship with health workers in the area.”* |
|  | 2b.  ✅ | Furthermore, it is very important for the authors to explain why they did a rural/urban selection of study sites, and not take it for granted. | Pg. 4, Line 196 | Linked to comment 2a, we have provided more detail on how the field-sites were chosen.  *“Fieldwork for the EXTRA study was conducted in two locations in South Africa, the urban township of Gugulethu and rural Bulungula. We deliberately chose two comparative field-sites, as we wanted to explore the perceived treatment burden of PLWMM in both rural and urban settings, as access to care, support networks and caregiving experiences may differ. In Gugulethu, we selected participants who were enrolled in a linked cross-sectional study that measures the prevalence, patterns and factors of multimorbidity in adults with HIV (3). The field-site in Bulungula was chosen due to an ongoing relationship with health workers in the area.”* |
|  | 2c.  ✅ | As the findings show, there appears to be a lot of differences in the data from these two study sites. Was there the need to have rural/urban study sites? Was it planned or not planned? Why was it necessary? This will go a long way to enrich the discussion of the findings. | Pg 4., (line 180-184) | *Have added the following text – line 180-184:*  *By design, this study aimed to comparatively explore the differences in treatment workload and caregivers’ experiences in two distinct settings where participants experience various degrees of precariousness, including lack of access to services, poverty, inadequate housing or social isolation. There is also a difference in prevalence and patters of multimorbidity between rural and urban context, and variations in self-rated health and work productivity [REFS]. Therefore, multimorbidity can have significant impact on both treatment burden and caregiving experience, which is further explored in this study.* |
| 3. Data management & analysis | 3a.  ✅ | Framework analysis--the authors need to explain, backed by the literature, what this analysis is, and why the need to us it, i.e. the advantages of using it. | Pg. 5 (Line 233-240) | We have rephrased the text, taking the comments into consideration.  *Before analysis commenced, MvP and NM transcribed and translated all audio-recorded interviews into English. Framework analysis guided the thematic analysis of data, widely used in health and social science research and is particularly suitable when researchers code and analyse data in a team (39, 40). We used framework as an abductive approach, using BoTT and its linked domains to guide our early interpretation of the data. After this, we moved on to thematic analysis to inductively analyse the data. This analysis was an interpretive process led by MvP and Charlotte Slome (CS), using the following steps: familiarisation, development of a coding framework, charting, and further mapping and interpretation (39).* |
|  | 3b.  Done | Ethical considerations: certain acronyms need to be explained/expanded for the first time: for example, BI, CHWs HCP, EPWP; and CHC. | Throughout | Thank you. We have corrected the text to ensure acronyms are explained and expanded on throughout the text. |
|  | 3c.  ✅ | Tables: The titles/headings for all the tables seem insignificant, based on the font sizes. The font sizes must be made equal to the font in the body of the paper, and each table must have its source and date of generation (e.g., 2022 added to the source). | Throughout the test | Font size has been changed to match body.  The four tables are new and have all been created for this paper, so we do not believe they need a source and date of generation. |
|  | 3d.  ✅ | Obviously, the first table does not fall under the subheading “Ethical considerations”. An appropriate subheading should be created for it or it should be moved and placed before the sub-section on ethical considerations. | Pg. 5, Line 244 | The table was moved under the data management & analysis section. |
| 4. Findings | 4a.  ✅ | In tables 2 and 3, and in the narrative before these, although you mention studying co-morbidities, and it is one of the basis for having the respondents (if I am not mistaken), several of the respondents have only one morbidity classifications (Hypertension or heart condition or heart disease, etc.). I am not sure how this ties in with the premise of studying co-morbidities. | Pg. 6-7 | All respondents were HIV+, so the comorbidities listed in the table were in addition to HIV. However, in response to another comment, we have decided to change the layout of the table to include both carer and patient demographics. The new table layout is formatted to have one column for ‘Conditions,’ which includes HIV and any other condition mentioned. |
|  | 4b.  ✅ | Page. 10: “As her mother was receiving a disability grant, she would often buy clothes and other necessities for her grandson and assist her daughter financially when possible.” The authors need to clarify if the funds used here is from her mother’s disability grant. It is not readily clear. | Pg. 10, Line 398 | Sentence was changed to:  *“Using money she received from her disability grant, the mother would buy clothes and other necessities for her grandson and assist her daughter financially when possible.”* |
|  | 4c.  ✅ | Before breaking the findings into the different kinds of support, it is necessary for the authors to give a summary/introduction that the data yielded three different kinds of support, before they detail the three. It reads better this way and prepares the reader to know what to expect. | Pg.6, line 362 - 267 | We have added the following text:  “The collected findings yielded five different themes in which support was provided; to navigate health services, emotional, logistical, financial and informational.” |
| 5. Discussion | 5a.  ✅ | Paragraph 1: there is the need for a more nuanced and a little more detailed rural-urban discussion of the findings than the little that appears at the end of this paragraph. | Pg.14, line 618 | We have provided more nuance in the discussion of the differences between urban and rural participants.  *“Patient participants in both the urban and rural field-sites needed support to manage their conditions and were grateful for the assistance carers provided. However, support was structured differently between the two field sites, especially practically, financially, and regarding access to care, whereby accessing health services was more time consuming for patients and their caregivers in the rural area. In Bulungula, carers sought additional support from the whole community, for example, when needing to find refuge on long journeys to the clinic, actively relying on the values of Ubuntu, an essential lifeline during health emergencies or food shortages (9, 10, 43, 44). In urban areas, the care networks were smaller, but caregivers provided more emotional support, which was hardly mentioned by rural carers (44).”*  Additional reference:  Mbokazi, Nonzuzo, Myrna van Pinxteren, Katherine Murphy, Frances S Mair, Carl R May, and Naomi S Levitt. “Ubuntu as a Mediator in Coping with Multimorbidity Treatment Burden in a Disadvantaged Rural and Urban Setting in South Africa.” Social Science & Medicine 334 (2023): 116190. |
|  | 5b.  ✅ | Paragraph 2: 3^rd^ sentence— “Younger carers, particularly adult children caring for parents, experienced opportunity….” In my mind the opportunity cost would be for every caregiver. This needs to be emphasised and then the authors can further highlight that of the younger caregivers. | Pg.15, Line 633-639 | Sentence has been changed to:  *“All carers experienced opportunity costs, but this was expressed most strongly by younger carers, particularly adult children caring for parents, who needed to navigate looking for employment while caring, which led to feelings of loneliness, especially when they were not able to rely on other family members (3, 25).”* |
|  | 5c.  ✅ | “For rural female carers, their role was not always voluntary, but rather a transactional appointment, due to paid lobola (bride price).”  This sentence seems a generalisation. Is it that no female carer in the urban area was married to the patient? Is it that all the female carers in the rural area were married to the sick persons? Is it that al female carers in the rural areas who had romantic relationships with the patient had had bride price paid for them? Is it in the rural areas only that married persons paid bride price, and not in the urban areas too, since the respondents from both communities belong to the same tribe. In this respect, does living in rural areas mean paying bride price and in the urban areas mean not paying bride price for marriage? These need to be carefully unravelled. | Pg. 15, discussion | Thank you, we agree. The sentence was removed because we don’t have a comparison between rural and urban in terms of the daughter-in-law, as we didn’t interview any in Cape Town. |
|  | 5d.  ✅ | “…This is unique, as socio-economic deprivation and the real-life impact of financial precarity on caring networks is underreported in the literature, especially in studies conducted in high income settings.”  At least two of the literature being referred to here needs to be cited to support this seemingly outstanding statement, to convince the reader. | Pg. 15, Line 640-645 | Thank you, we have changed the text and added the references as per your suggestion.  *“Commonly prioritized in other informal caregiving papers is the emotional workload of caregivers, followed by social and financial burdens (42). In this study, caregivers struggled most with the financial workload and the impact of precarity on their caregiving capacity. This is unique, as socio-economic deprivation and the real-life impact of financial precarity on caring networks is under acknowledged in the literature, especially in studies conducted in high income settings*  van Pinxteren, M., N. Mbokazi, K. Murphy, F. S. Mair, C. May, and N. Levitt. "The Impact of Persistent Precarity on Patients' Capacity to Manage Their Treatment Burden: A Comparative Qualitative Study between Urban and Rural Patients with Multimorbidity in South Africa." [In eng]. *Front Med (Lausanne)* 10 (2023): 1061190. <https://doi.org/10.3389/fmed.2023.1061190>.  McKee, Martin, Aaron Reeves, Amy Clair, and David Stuckler. "Living on the Edge: Precariousness and Why It Matters for Health." *Archives of Public Health* 75, no. 1 (2017): 1-10.  Hill K, Hirsch D, Davis A. The Role of Social Support Networks in Helping Low Income Families through Uncertain Times. Social Policy and Society. 2021;20(1):17-32. doi:10.1017/S1474746420000184 |
|  | 5e.  ✅ | The first part of the discussion, up to the end of the second paragraph, seems to repeat the findings, without doing due diligence of shedding more light on how the literature quoted in these paragraphs shed light on their findings—e.g. compare, contrast with, support theirs etc. This needs to done, to better inform the reader. | Pg. 15, Line 631 – 648 | We have now added several references which link our findings to the larger body of literature – debating the similarities and differences between caregiving experiences in LMICs and high-income settings, between female and male caregivers and the representation of informal caregiving in current global health debates.  We have put more emphasis on the rural and urban comparison in the first paragraph, linking the findings to the larger body of literature in the second paragraph.  *“Our findings add to the small body of literature exploring informal caregiving in low-middle income contexts and are consistent with previous results (3, 7, 25, 44, 45). Like other settings, a majority of caregivers were female and were most often the spouse or adult child. All carers experienced opportunity costs, but this was expressed most strongly by younger carers, particularly adult children caring for parents, who needed to navigate looking for employment while caring, which led to feelings of loneliness, especially when they were not able to rely on other family members (3, 25). Female caregivers experienced the largest workload, as they are culturally expected to fulfil caregiving duties, leading to opportunity costs such as cutting back on paid work or being unable to combine care work with seeking employment (46). In our study, younger women expressed the emotional strain of their caring responsibilities (25, 46). Commonly prioritized in other informal caregiving papers is the emotional workload of caregivers, followed by social and financial burdens (43). In this study, caregivers struggled most with the financial workload and the impact of precarity on their caregiving capacity. This is unique, as socio-economic deprivation and the real-life impact of financial precarity on caring networks is under acknowledged in the literature, especially in studies conducted in high income settings (27, 47). The wider literature also largely explores the workload of caregiving when caring for patients with short-term, high-demand conditions such as cancer or end-stage organ failure that requires palliative care , whereas our study highlights the underrepresented cohort of caregivers caring for people with long-term, low care needs conditions (13, 48, 49).”* |
|  | 5f.  ✅ | Table heading/titles must uniformly come at the bottom or above the table. Table four has its at the bottom, while the rest do not. | Throughout | Table headings have been corrected and moved to the top. |
|  | 5g.  ✅ | 3rd paragraph: First sentence  “Using the BoTT as a framework throughout all phases of this study enabled us to explore how informal caregiving is operationalised in rural and urban South African settings.”  The highlighted part of the sentence seems a generalisation. The study did not use random samples, and it was restricted to about 2 Provinces, and within these, purposive sampling was used to select the respondents—not even the study sites: hence the findings cannot be generalised to the whole of SA, not even study Provinces, and even the study communities. Conclusions can only be made regarding the respondents in the study, in this case. This correction will have to be done. | Pg. 15, Line 667 | Thank you for pointing this out. We have rephrased the sentence to reflect:  *“Using the BoTT as a framework throughout all phases of this study enabled us to explore how informal caregiving is operationalised in our rural and urban South African study sites.”* |
|  | 5h.  ✅ | 4th paragraph, line 2: “…the most stressing for informal…” “…most stressful for…” instead? | Pg. 16, 686 | We agree. Stressing has been changed to stressful. |
|  | 5i.  ✅ | 5th paragraph: the first two sentences in the paragraph, beginning with “Secondly, during...” seem to belong to the findings section. They were never raised in the findings section, and yet they get discussed. I presume the discussion section is to cover issues raised in the findings, and not to turn the discussion into another section where new findings are provided. The authors should consider finding a more encompassing subtopic for reporting the other findings they leave out at the section on findings and report in the discussions. This is, if they find it important to report these additional findings. Else they can leave them out at the section on discussion as well, for now. They may capture these in another paper, etc. | Pg. 16, Discussion | The first sentence, referencing COVID-19 has been taken out, as it was not mentioned in the findings. However, the second sentence discussing how carers felt underinformed was stated in the findings (page. 13, line 622).  An additional sentence was added to the findings on pg. 12 (line 594) to further clarify:  *“Information shared through radio and television programmes often focused on communicable diseases, such as HIV, TB, and COVID-19.”* |
|  | 5j. | 6th paragraph: “Thirdly, caregivers in urban areas felt excluded from health services…” The same point expressed immediately above seems true about this statement.  “Additionally, health workers should be encouraged to create long-term relationships with informal carers, as they can be allies in caregiving.”  It will be very important for the authors to add information on how this could be done—some suggestions of the ‘how to’ will be great. This is to make this recommendation more practical, as the ‘how to’ does not seem to be easy. | Pg. 16, Lines 761 | The following has been added to the text to clarify how to operationalise this recommendation:  “Additionally, caregivers can be allies to health workers when there is a long-term relationship between health workers, PLWMM and informal carers. To foster this, low staff turnover is key and health workers should ask caregivers to join consultations. During clinic visits, caregivers should be encouraged to ask questions and receive emotional support from health workers.” |
| 6. Others | 6a.  ✅ | The text must make reference to the respective tables. |  | All the tables are mentioned at least once in the text of the paper.  Table 1 – pg. 5 (Line 286)  Table 2 & 3 – pg. 6 (Line 318)  Table 4 – pg. 14 (Line 751) |
|  | 6b✅ | First sentence under limitations has [REFS]. Not sure if this meant references were to be added. If that is the case, then they need to be provided. | Page 16, line 717 | Thank you, the mistake has been corrected.  Cornell, Morna, Leigh F Johnson, Robin Wood, Frank Tanser, Matthew P Fox, Hans Prozesky, Michael Schomaker*, et al.* "Twelve‐Year Mortality in Adults Initiating Antiretroviral Therapy in South Africa." *Journal of the International AIDS Society* 20, no. 1 (2017): 21902. |

| Reviewer 2 comments | | | | |
| --- | --- | --- | --- | --- |
| Comment | |  | Corrected/Revised | |
| 1. Overall | 1a.  ✅ | I would suggest rethinking the use of the two analytic lenses. Perhaps remove reference to Cumulative complexity model as you don’t seem to have used it in the analysis.  Burden of Treatment theory (BoTT) extends the cumulative complexity model anyway so it seems redundant to include both. BoTT talks about capacity components of agency, relationality, control and opportunity which aren’t evident in the framework or the results.  Consider revisiting the data analysis using the BoTT components. | Throughout the text | We have removed the reference to the CuCoM model, and emphasized the use of the BoTT throughout the text, mainly in the data analysis and findings section, including in Table 4 in the discussion. |
|  | 1b. | There is minimal use of the patient data. I think it would be clearer if you referred to your 16 carers as your participants. | Throughout the text | References to patients has been changed throughout the paper to ‘patient participants’ to better distinguish between patients and carers. Carers are defined as ‘carers’. |
|  | 1c. | Your table of participants should be the CU IDs, and their demographics. Then link to the patient (for whom they care) demographics. If you want to include patient data, it needs to be much clearer, which patient data was used and why and how it was used. | Pg. 6, 7 | The table of participants, Tables 2 and 3, have been reworked to include carer study IDs, linked to the patient ID. |
| 2. Abstract | 2a. | Review background and methods for consistency with final papers actual objectives. The result “feeling a duty to care eased the perceived carer burden” is not present in your results and seems a bit incongruous. The first two lines in conclusion are probably results. | Pg. 2, line 52 – 81 | We have rewritten the abstract, based on the provided comments.  Background:  The importance of informal caregiving for chronic illness has been well established in African contexts but is underexplored in the context of HIV/NCD multimorbidity, particularly in South Africa. Building on treatment burden theories that investigate the workload in the context of chronic illness this paper explores how informal caregiving networks increases the capacity people living with multimorbidity (PLWMM) in low-income settings in urban and rural South Africa.    Methods:  Qualitative semi-structured interviews were carried out with thirty people living with multimorbidity and sixteen informal caregivers between February and April 2021 in two settings, Cape Town (urban) and Bulungula (rural). Interviews were transcribed and data analysed both inductively and deductively using framework analysis, hereby, building on the principles of the burden of treatment theory (BoTT) as a theoretical lens.    Findings:  Our findings show that informal carers provided different types of support to people living with multimorbidity, including emotional, logistical, health services and informational support to ease the patient’s treatment burden. Additional support networks, access to grants or financial security, and feeling a duty to care eased the perceived carer burden while a lack of social support, financial hardship and insufficient information hindered their capacity to support others. Overall, informal caregiving increases the capacity of people living with multimorbidity in low-income settings in South Africa.    Discussion:  Informal caregiving in the context of multimorbidity is structured through social obligations, kinship connections, cultural expectations, and an individual sense of agency. Carers, even when committed to assist, also experience caregiving opportunity costs, which are rarely addressed in the literature. By proposing interventions that can actively support informal caregivers, we can begin to develop solutions that can optimize the role of informal care networks, with a view to improve the health-outcomes PLWMM in South Africa. |
| 3. Introduction | 3a.  ✅ | The fourth paragraph, regarding theory, would be better in methods. | Pg. 5, line 236 – 240 | We think an introduction to a BoTT is necessary, as it was the theoretical basis through which we based our work. However, we have expanded on the BoTT and its use in the analysis in the Methods section.  *We used framework as an abductive approach, using BoTT and its linked domains to guide our early interpretation of the data. After this, we moved on to thematic analysis to inductively analyse the data.* |
|  | 3b.  ✅ | Suggest remove reference to Cumulative complexity model. | Pg. 3 | Deleted. |
|  | 3c.  ✅ | The fifth paragraph might be better in discussion. The final two lines are the aims of the paper- | Pg. 15, line 649 -633 | We have moved this complete paragraph to the discussion:  *As South Africa is developing new, integrated primary care systems to improve health service provision for people with chronic illnesses, the role of informal caregiving for those living with multimorbidity is becoming increasingly important, emphasising the ability for patients to self-manage their conditions (27). Based on the WHO’s Care of Chronic Conditions (ICCC) framework, the National Department of Health is rolling out the Integrated Chronic Disease Model (ICDM) in 42 primary care clinics in three of nine provinces (27). Although still being operationalized, early evaluation studies show that the ICDM fails to address organisational issues, offers insufficient self-management support for patients, and is insensitive towards the increased illness burden faced by people living with multimorbidity (28, 29). Furthermore, while both ICDM and ICCC models recognise patients’ family as stakeholders, no attention is being given to how their role can be better operationalised or supported when providing care for people living with multimorbidity. This is problematic, as initiatives such as the ICDM consider family members to be semi-formalized caregivers who are now responsible for the bulk of the ‘chronic homework’ stipulated by the health system (30). Findings in this paper shed light on how the support or informal caregivers is structured and underscores how their involvement increases the capacity of PLWMM.* |
|  | 3d.  ✅ | It would be better to leave the details about EXTRA to the methods. The aim of the study is to describe the types of support informal caregivers provide to people living with HIV/NCD multimorbidity in South Africa. | Pg. 4 , line 158 - 163 | Thank you, we have changed the sentence to move mention of the EXTRA study. It now reads as follows:  *As informal caregiving in the context of multimorbidity is currently underreported, this paper explores various types of support provided by caregivers to people living with HIV/NCD multimorbidity in South Africa. We also aim to understand the impact of the context of poverty, rurality, and gender roles on the ability of caregivers to improve capacity of the patient. Using these insights, we then describe ways to better incorporate support networks in an integrated care system whilst increasing the capacity of both patients and caregivers* |
|  | 3e.  ✅ | I think you also attempt to understand the impact of the context (poverty, rurality, gender roles etc) on the ability of caregivers to improve capacity of the patient, and provide recommendations to support caregivers in this activity. Such an aim would be consistent with BoTT. | Pg.4, Line 160-163 | The following has been added to introduction:  *We also aim to understand the impact of the context of poverty, rurality, and gender roles on the ability of caregivers to improve capacity of the patient. Using these insights, we then describe ways to better incorporate support networks in an integrated care system whilst increasing the capacity of both patients and caregivers* |
| 4. Methods | 4a.  ✅ | There is a degree of repetition and confusion in the presentation of the methods. Your own paper on the methodology is structured much better. Use that as a guide in addition to comments. Suggest a structure like below… | Pg. 4 | Thank you, we have used your suggested structure to adjust the section. A separate section for ‘setting and recruitment’ has been added.  The section has also been re-read and adjusted to reduce repetition. |
| 5. Findings | 5a. | Table 2 and 3 are the participant of EXTRA. For your substudy please show the participants as the carers (plus any patient data you chose to include – with justification in methods). This will resolve the problem of your quote IDs not being present in the table. Eg CU and CR numbers | Pg. 6-7 | Thank you, we agree. The tables have been reformatted to include both carer and patient participant demographics. |
|  | 5b.  ✅ | The findings are structured according to the 4 activities that carer’s do. There is little to reflect BoTT. Suggest restructure your analysis around BoTT with a much shorter section on activities done. Eg Table 4, second column, attributes impacting on carer’s capacity could be in results. However I think in BoTT you should be talking about attributes impacting on carer’s ability to support patient capacity. | Pg. 6 onwards | Taking into consideration this feedback, we have incorporated the terminology used in the BoTT in both the findings section and table 4, highlighting the links between patient and carer capacity and how this is represented within the theoretical framework. Changes have been made throughout the findings section – Starting on page 6, the beginning of the Findings section. |
|  | 5c.  ✅ | There is limited comparison of urban and rural – suggest strengthen this if possible (or if not possible remove the wording about comparing in other sections of the paper. | Throughout the text | We believe the paper offers an in-depth comparison of urban and rural, both in contexts and experiences of access to healthcare as well as support provided by informal caregivers. We have expanded on the differences and similarities between the settings to show the varieties of experiences in South Africa, and further unpacked this in the Discussion section. Changes can be found on:  Pg. 9, line 339-340: “In Bulungula, many carers were responsible for keeping track of patient participants’ appointment dates, as most rural participants were illiterate.”  Pg. 9, line 355: “However, emotional support and the emotional aspects of caregiving were more widely acknowledged in urban dyads.”  Pg. 11, line 477: “Accessing for diversity of food was more challenging for rural participants compared to urban as they could not readily access supermarkets.”  Pg. 12, line 527: “Financial precarity was more prevalent in the rural areas…”  Pg. 12, line 531: “Due to the distances between amenities in the rural areas, the costs associated with traveling to the clinic or hospital were far greater compared to the urban areas.” |
|  | 5d.  ✅ | The part on Providing emotional support – seems to be more about emotional impacts of being a carer on the carer. If you re-organise the analysis to focus on attributes impacting on carer’s ability to support patient capacity, the content is appropriate. | Pg. 9 | Several sentences that focused more on the patient participants were deleted to provide clarity. |
| 6. Discussion | 6a.  ✅ | The discussion first paragraph includes a few findings that are of interest but are unclear in your results   1. the role of obligation (and see also comment about abstract) 2. patient loneliness if they didn’t have a carer (not sure if this is relevant in your study) 3. Rural support networks (could be much stronger in results). | Pg. 13 and throughout the text (pages and lines indicated in the table) | 1. Social obligation is mentioned in the findings (pg.9, 364-366): *“Although often described as logistically challenging and time-consuming, they also felt proud of their caring abilities. This social obligation to care was expressed mostly by family members.”* 2. In findings: *“All carers experienced opportunity costs, but this was expressed most strongly by younger carers, particularly adult children caring for parents, who needed to navigate looking for employment while caring, which led to feelings of loneliness, especially when they were not able to rely on other family members.”*   We have further emphasized in the discussion section that providing emotional support for carers might have the potential to increase their capacity to care and in turn, improve the wellbeing of PLWMM. From **Page 16, line 729 …. Plus table 4.**   3. *Added on page 15:* These findings point to a difference in social cohesion between urban Gugulethu and rural Bulungula, whereby rural carers and patient participants could rely more easily on their extended networks, whereas some carers and patient participants in Gugulethu felt socially excluded. |
|  | 6b.  ✅ | The second paragraph regarding gender roles is interesting but not very evident in the result. If you redo your table of participants as the carer and include their gender and age it would be useful to support this part of the discussion. | Pg. 14 | Tables 2 and 3 have been reworked to include carer gender and age to support this claim. |
|  | 6c.  ✅ | The discussion affirms the suitability of BoTT, perhaps without justification, given its limited use in the results and earlier discussion. BoTT talks about capacity components of agency, relationality, control and opportunity which aren’t evident in the results. | Throughout the findings and discussion section | As mentioned in the response to comment 5b, we have made substantial changes in the findings section to link carers experiences to the different components of the BoTT, hereby highlighting the complexity between individual agency, social capital, functional performance and capacity. |
|  | 6d.  ✅ | The interventions are good and would be stronger if the results included the attributes impacting on carer’s ability to support patient capacity. | Pg. 17 | As per the reviewer’s suggestion, we have updated the table 4 to reflect how the proposed interventions can increase patient and carer capacity. |
|  | 6e.  ✅ | Page 18 – “we only recruited a few male caregivers” – how many should be clear in results | Pg. 16 | The wording has been changed from ‘a few’ to ‘four.’ |
|  | 6f.  ✅ | “A key strength is the comparative nature of the study” – need to strengthen comparisons to make this claim. | Pg. 18, line 759 | We believe we have strengthened the comparisons throughout the paper. The sentence has been adjusted slightly to be more specific and now reads:  *A key strength is the comparative nature of the study, allowing us to explore the variation within a common experience, as patient participants and carers’ lived experiences differed based on their physical access to care, links to the community, and caring burden. However, there were many similarities between caregiver’s experiences, regardless of where they lived.* |
|  | 6g.  ✅ | “reached data saturation’ – how this was determined should be in methods. | Pg. 18, line 763 - 765 | This has been deleted from the sentence. The full sentence now reads:  *“Another strength is the prolonged engagement with the study sites, as we travelled regularly to Gugulethu CHC and stayed in Bulungula for two weeks and were able to collect rich insights on people’s perceived treatment burden and capacity.”* |
| 7. Minor issues | 7a.  ✅ | Figures/Tables – Table titles should be at the top of the table |  | Table titles have been corrected. |
|  | 7b.  ✅ | Reference missing end of page 17. | Pg. 16 (748) | The following reference has been added:  Oni, Tolu, Nuala McGrath, Rhonda BeLue, Paul Roderick, Stephen Colagiuri, Carl R May, and Naomi S Levitt. "Chronic Diseases and Multi-Morbidity-a Conceptual Modification to the Who Iccc Model for Countries in Health Transition." BMC public health 14, no. 1 (2014): 1-7. |
|  | 7c.  ✅ | Missing “and” in first sentence of discussion. | Pg. 13 | ‘and’ has been added to the sentence. |
|  | 7d. | Overall a little lengthy, could be more succinct. |  | We have made the paper more succinct where possible. |
