# Supplementary material for: Exploring the workload of informal caregiving in the context of HIV/NCD multimorbidity in South Africa
Source: PLOS Glob Public Health. 2024 Oct 8;4(10):e0003782. doi: 10.1371/journal.pgph.0003782 (PMC11460685; doi:10.1371/journal.pgph.0003782)
Supplement: Attachment — Submitted filename: PLOS REVISIONS DRAFT 2 - 17062024.docx [file pgph.0003782.s004.docx]

**REVISIONS ROUND 2**

**Thank you so much for your additional comments, and taking the time to review this paper. Below, you can find the table of changes with the responses to comments.**

**Examiner 2:**

| **Comment** |  | **Corrected/Revised** |
| --- | --- | --- |
| 1. | **In Methods** “Setting and recruitment” doesn’t include details of recruitment. I suggest the section from study design could be second paragraph in setting and recruitment. “Patient participants were selected using a purposive sampling method with the following criteria: adults living with HIV and at least one other chronic condition who attended public primary health care services. Patient participants were able to bring their primary caregivers, who were interviewed as carer participants, but those without a caregiver present were not excluded from the EXTRA study. Carer participants referenced in this study were either present with the patient participant or mentioned by the patient participant during the interview.” This could be followed by this section from data collection. “In Cape Town, 16 patients and 10 ……with the Primary Health Care system (37, 38)” | Please see below the corrected text as recommended – line 187 – 206, page 4.  *“Fieldwork for the EXTRA study was conducted in two locations in South Africa, the urban township of Gugulethu and rural Bulungula. We deliberately chose two comparative field-sites, as we wanted to explore the perceived treatment burden of PLWMM in both rural and urban settings, as access to care, support networks and caregiving experiences may differ.*  *Patient participants were selected using a purposive sampling method with the following criteria: adults living with HIV and at least one other chronic condition who attended public primary health care services. Patient participants were able to bring their primary caregivers, who were interviewed as carer participants, but those without a caregiver present were not excluded from the EXTRA study. Carer participants referenced in this study were either present with the patient participant or mentioned by the patient participant during the interview. The interview guide was adapted from the Burden of Treatment questionnaire (BTQ), and BoTT, aiming to explore patient and carer experiences (23). An in-depth description of the methodological considerations is published elsewhere (26).*  *In Cape Town, we selected participants who were enrolled in a linked cross-sectional study that measures the prevalence, patterns and factors of multimorbidity in adults with HIV (32). Here, 16 patient participants and 10 informal carers were recruited at a community health clinic (CHC) through a trusted fieldworker who also facilitated the consent process The field-site in Bulungula was chosen due to an ongoing relationship with health workers in the area. Here, 14 patients and 6 informal carers were recruited from two villages and were receiving treatment at various primary health clinics in the area.”* |
| 2. | The following line in study design would be better in data collection (replacing section removed as above). “The interview guide was adapted from the Burden of Treatment questionnaire (BTQ), and BoTT, aiming to explore patient and carer experiences (23)” | Added to Data collection section – Line 224 – 227, page 4.  The interview guide was adapted from the Burden of Treatment questionnaire (BTQ), and BoTT, aiming to explore patient and carer experiences (23). An in-depth description of the methodological considerations is published elsewhere (26). |
| 3. | **Findings** Table has 15 participants in each site, but your findings say “16 in Gugulethu, Western Cape and 14 in Bulungula, Eastern Cape” Table 2 patient ID – I think these should be PU not PR numbers? | We have rectified the text about recruitment of participants on page 4 (line 199 – 204), and updated the details of the participants in Table 2 and 3, on page 6 & 7.  *“Here, 16 patient participants and 10 informal carers were recruited at a community health clinic (CHC) through a trusted fieldworker who also facilitated the consent process The field-site in Bulungula was chosen due to an ongoing relationship with health workers in the area. Here, 14 patient participants and 6 informal carers were recruited from two villages and were receiving treatment at various primary health clinics in the area.”* |
| 4. | I like the reformatting of the table to match carers to participants but where there is no Carer interview, it feels a bit misleading to list a CR or CU. I suggest just greying out the CR or CU squares if there is a no carer interview. Also I am unclear what you mean by “dyadic interview”? I thought it was that the carer was interviewed – but you only have 6 yeses in Table 2. What type of interview did the other 4 carers do and can it be indicated on the table? | We have changed Table 2 and 3 to clarify whether the participants’ caregivers were interviewed – indicating ‘Carer Present’ with YES or NO. Where NO was applicable, we have added a grey colour to make the table easier to read. In the urban field-site, there were two patient participants who did not have a caregiver/support network, which is indicated in the table. |
| 5. | Eg CU10 – has quotes so was obviously interviewed but there is a no in “dyadic interview” column. | Apologies, this was an oversight on our part. We have no updated Table 2 reflecting the specifics of CU010. |
| 6. | L373 – the quote doesn’t seem to match the findings – loneliness? | Have revised the section for consistency and explained the link between carer capacity and belief systems. This part precedes the part about emotional strain on caregivers, where we have replaced the word loneliness with ‘exhaustion’, as this explains the carer’s feelings better.  Page 10, line 366 - 382  Other carers would rather take the caring responsibility themselves, even when they would live near other family members.  *"I do not even call other people to help me, like his sister who lives in one of the back rooms. I do it all myself. Sometimes, they will hear later that he was not well. But there is no need to tell her because she probably would not help me anyway. I do it all myself without a problem.”* (CU010F, girlfriend)  This carer ascribed her caring abilities to both her personality, but also attributed her caring capacity to her belief system. She relied on God to give her strength.    *"I think I was given the care by God. I never get tired of someone who is sick or get impatient about looking after them. God gave me the ability to care."* (CU010F, girlfriend) |
|  | **Journal requirements** |  |
| 1. | 1. Please review your reference list to ensure that it is complete and correct. If you have cited papers that have been retracted, please include the rationale for doing so in the manuscript text, or remove these references and replace them with relevant current references. Any changes to the reference list should be mentioned in the rebuttal letter that accompanies your revised manuscript. If you need to cite a retracted article, indicate the article’s retracted status in the References list and also include a citation and full reference for the retraction notice. | All references were checked for accuracy. |
| 2. | We have noticed that you have uploaded Supporting Information files, but you have not included a list of legends. Please add a full list of legends for your Supporting Information files after the references list. | Legend for appendix included after the reference list |
