# Supplementary material for: Exploring the workload of informal caregiving in the context of HIV/NCD multimorbidity in South Africa
Source: PLOS Glob Public Health. 2024 Oct 8;4(10):e0003782. doi: 10.1371/journal.pgph.0003782 (PMC11460685; doi:10.1371/journal.pgph.0003782)
Supplement: Attachment — Submitted filename: Response to the reviewers 21 July 2024.docx [file pgph.0003782.s005.docx]

Reviewer comments

|  | Original page (line) | Reviewer comment | New page (line) | Author comment |
| --- | --- | --- | --- | --- |
| 1 | Page 6 (259-263) | You haven’t fully addressed an inconsistency in the number of patients/carers. The text indicates 16 patients in Gugulethu and 10 carers but your table 2 has 15 patients and 11 carers. Please rectify. | Page 6 (259-263) | Table 2 was missing a participant, the error has now been rectified and PU007 has been added to the table. The text now matches the table, with 11 informal caregivers and 16 patients in Gugulethu. |
| 2 | Page 7-8 (269; 273) | NA in the tables 2 and 3 is better as N/A. In the table key it should be N/A = Not available | Page 7-8 (269; 273) | The key in both tables has been changed to say:  N/A= Not available |
| 3 | Page 7-8 (269; 273) | Grey = not present for interview/no caregiver identified would be better as not present for interview OR no caregiver identified | Page 7-8 (269; 273) | We have amended the sentence to say:  Grey= Not present for interview OR no caregiver identified |
| 4 | Page 20 (876);  Page 22 (937) | 27 and 51 appear to be the same paper – same DOI. Please rectify. | Page 20 (876);  Page 22 (937) | This paper was duplicated. We have removed 51 as a citation and changed the in-text citation on page 15 (line 659) to 27. |
