# Supplementary material for: Exploring the workload of informal caregiving in the context of HIV/NCD multimorbidity in South Africa
Source: PLOS Glob Public Health. 2024 Oct 8;4(10):e0003782. doi: 10.1371/journal.pgph.0003782 (PMC11460685; doi:10.1371/journal.pgph.0003782)
Supplement: Attachment — Submitted filename: Response to Reviewers 07082024.docx [file pgph.0003782.s006.docx]

Editor comments

|  | Original page (line) | Reviewer comment | New page (line) | Author comment |
| --- | --- | --- | --- | --- |
| 1 | N/A | Please include a complete copy of PLOS’ questionnaire on inclusivity in global research in your revised manuscript…  Please upload a completed version of your questionnaire as Supporting Information when you resubmit your manuscript. | Pg. 6 line 257 | We have included the questionnaire. As per the questionnaire guidelines, we have also added a sub-section to the Methods section, to state:  Inclusivity in global research  Additional information regarding the ethical, cultural, and scientific considerations specific to inclusivity in global research is included in the Supporting Information (Appendix 2). |
| 2 | N/A | Please review your reference list to ensure that it is complete and correct. If you have cited papers that have been retracted, please include the rationale for doing so in the manuscript text, or remove these references and replace them with relevant current references. | Pg. 21 line 920 | Thank you. We did not identify a retracted article; however, we did identify one article (Reference #45) in which an author’s name has been revised. The reference has been amended to state:  45. Thrush A, Hyder A.A. The neglected burden of caregiving in low-and middle-income countries. Disability and health journal. 2014;7(3):262-72. |

Reviewer comments

|  | Original page (line) | Reviewer comment | New page (line) | Author comment |
| --- | --- | --- | --- | --- |
| 1 | Pg. 6 line 268 | Note - The box for CU007 has not been greyed in the same way as CU012. | Pg. 6 line 268 | Thank you, the shading has been corrected for this. |
| 2 | Pg. 6 line 268 | For N/A - the NA in the table should also be changed to N/A, as well as the in the text below the table. It would be worth confirming that for conditions you mean N/A - or if you mean none - as for CU002 - then write none. | Pg. 6 line 268 | Thank you. We have amended the tables to change all NA’s to N/A. For clarity, N/A means we did not get that information during the interview, whereas, ‘None’, as stated for CU002, means they have no medical conditions. |
