# Supplementary material for: Exploring the workload of informal caregiving in the context of HIV/NCD multimorbidity in South Africa
Source: PLOS Glob Public Health. 2024 Oct 8;4(10):e0003782. doi: 10.1371/journal.pgph.0003782 (PMC11460685; doi:10.1371/journal.pgph.0003782)
Supplement: Attachment — Submitted filename: Response to reviewers 27082024.docx [file pgph.0003782.s007.docx]

**Response to reviewers:**

Thank you for giving us the chance to revise this paper. As suggested, we have added a sentence to the table with participants, to explain why CR007 was excluded: *'Excluded from study, as the patient participant did not meet the inclusion criteria of HIV/NCD multimorbidity'*.

We have also uploaded the supporting materials, namely; Appendix 1: Interview schedule EXTRA FINAL DRAFT 30102023, and Appendix 2: Inclusivity in global research questionnaire in the system, and added the legend in the paper.
